# Baicalin Ameliorates Experimental Liver Cholestasis in Mice by Modulation of Oxidative Stress, Inflammation, and NRF2 Transcription Factor

**DOI:** 10.1155/2017/6169128

**Published:** 2017-07-05

**Authors:** Kezhen Shen, Xiaowen Feng, Hao Pan, Feng Zhang, Haiyang Xie, Shusen Zheng

**Affiliations:** ^1^Key Laboratory of Combined Multi-Organ Transplantation, Ministry of Public Health, the First Affiliated Hospital, College of Medicine, Zhejiang University, Hangzhou, Zhejiang 310003, China; ^2^Collaborative Innovation Center for Diagnosis and Treatment of Infectious Diseases, the First Affiliated Hospital, College of Medicine, Zhejiang University, Hangzhou, Zhejiang 310003, China; ^3^Department of Urology, the First Affiliated Hospital, College of Medicine, Zhejiang University, Hangzhou, Zhejiang 310003, China; ^4^Division of Hepatobiliary and Pancreatic Surgery, the First Affiliated Hospital, Zhejiang University School of Medicine, Hangzhou, Zhejiang 310003, China

## Abstract

Experimental cholestatic liver fibrosis was performed by bile duct ligation (BDL) in mice, and significant liver injury was observed in 15 days. Administration of baicalin in mice significantly ameliorates liver fibrosis. Experimental cholestatic liver fibrosis was associated with induced gene expression of fibrotic markers such as collagen I, fibronectin, alpha smooth muscle actin (SMA), and connective tissue growth factor (CTGF); increased inflammatory cytokines (TNF*α*, MIP1*α*, IL1*β*, and MIP2); increased oxidative stress and reactive oxygen species- (ROS-) inducing enzymes (NOX2 and iNOS); dysfunctional mitochondrial electron chain complexes; and apoptotic/necrotic cell death markers (DNA fragmentation, caspase 3 activity, and PARP activity). Baicalin administration on alternate day reduced fibrosis along with profibrotic gene expression, proinflammatory cytokines, oxidative stress, and cell death whereas improving the function of mitochondrial electron transport chain. We observed baicalin enhanced NRF2 activation by nuclear translocation and induced its target genes HO-1 and GCLM, thus enhancing antioxidant defense. Interplay of oxidative stress/inflammation and NRF2 were key players for baicalin-mediated protection. Stellate cell activation is crucial for initiation of fibrosis. Baicalin alleviated stellate cell activation and modulated TIMP1, SMA, collagen 1, and fibronectin in vitro. This study indicates that baicalin might be beneficial for reducing inflammation and fibrosis in liver injury models.

## 1. Introduction

Chronic liver disease prevalence is increasing globally, and liver fibrosis associated with such chronic liver disease is an important cause of morbidity and mortality in the world [1]. Due to repetitive injury and inflammation in chronic liver disease, significant extracellular matrix is deposited and causes severe liver damage and organ failure. This liver fibrosis can also lead to cirrhosis and hepatocellular carcinoma [1]. Chronic cholestatic liver disease is also observed after liver transplantation [2]. Unfortunately limited antifibrotic therapies are available to treat patients with liver fibrosis [3, 4]. Many plant-derived flavonoids including baicalin show antifibrotic properties in the liver of animal models, but the mechanistic studies are limited.

The proposed mechanism of liver fibrosis includes oxidative tissue injury, mitochondrial dysregulation, and inflammation, which lead to apoptotic and necrotic cell death [5, 6]. Liver fibrogenic cells (myofibroblasts), mainly derived from hepatic stellate cells (HSC), play a central role during liver fibrosis [7]. At early stage of tissue injury, all surrounding cells to HSC contribute to its activation. Inflammation-mediated hepatocyte cell death and its engulfment by HSC result in a profibrogenic response and also activate Kupffer cells [8, 9].

**Figure 1:**
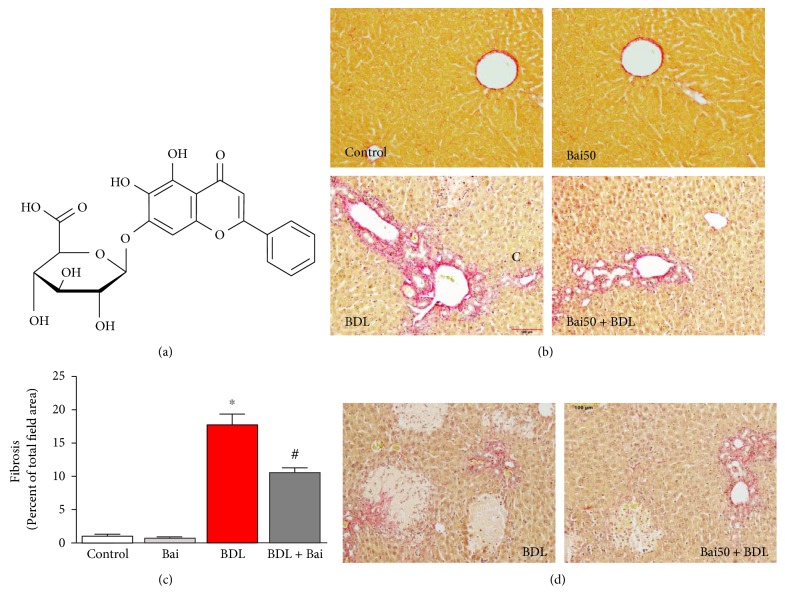
Baicalin significantly attenuated BDL-induced liver fibrosis in mice. (a) Chemical structure of baicalin. (b) BDL-induced liver fibrosis as measured by picrosirius red staining. The images were quantified in a blinded manner by ImageJ software (c). BDL resulted in severe liver fibrosis which was attenuated by baicalin treatment. Results are mean ± S.E.M. *n* = 6 for each group. ^∗^*p* < 0.05 versus control and ^#^*p* < 0.05 versus BDL. (d) Representative images for highlighting necrosis area in BDL and BDL samples pretreated with baicalin.

Flavonoids are natural compounds and are of considerable interest in the field of drug discovery as well as food supplement industry [10]. Baicalin is one such flavonoid and derived from the roots of *Scutellaria baicalensis* Georgi, and these plants are mainly cultivated in China, Siberia, Russia, and Mongolia [11]. The chemical nomenclature (IUPAC) is 5,6-dihydroxy-4-oxo-2-phenyl-4H-chromen-7-yl *β*-D-glucopyranosiduronic acid (Figure 1(a)). Protective effects of baicalin in many liver disease animal models have been reported. In a rat ischemia/reperfusion injury model, baicalin protects the liver by antioxidant and anti-inflammatory properties [12]. In concanavalin A-induced mouse liver injury, baicalin protects by modulating cytokine production [13]. Baicalin attenuates alcoholic liver injury involving sonic hedgehog pathway in addition to its antioxidant and anti-inflammatory properties [14]. In a high-fat diet-induced obesity model of mice, baicalin attenuates liver dysfunction by inhibiting CaMKK/AMPK/ACC pathway and protein kinase B/glycogen synthase kinase 3 beta pathway [15, 16]. Recently, baicalin is shown to protect the carbon tetrachloride-induced liver injury model of the rat by activating PPAR*γ* and inhibiting TGF*β*1 [17]. Here, we demonstrated the protective effect of baicalin in a cholestatic mice model was mediated via NRF2 transcription factor and its downstream target in addition to modulation of oxidative stress-generating enzymes and inflammation.

## 2. Materials and Methods

### 2.1. Mice Surgeries and Baicalin Treatment

Animal Ethics Review Committees of Zhejiang University approved the animal study protocol. Male inbred C57BL/6 (H2b) mice at 6–8 weeks old were obtained from the Animal Research Institution of Zhejiang Province (Hangzhou). BDL surgeries were performed under anesthesia as described earlier [5]. To determine the effect of baicalin on liver fibrosis, mice received one dose of preoperative (2 hours) IP injection of baicalin and alternate day postoperative IP injection of baicalin (50 mg/kg/day) for additional 14 days. For all animal experiments, we used 12 mice/group.

### 2.2. Liver Fibrosis Marker

Quantitative determination of liver fibrosis was performed with Picrosirius Red Stain kit (Abcam Company Ltd., China) as described earlier [5]. The quantification of collagen staining (red) was analyzed from images using ImageJ software (NIH, USA).

### 2.3. mRNA Quantification

RNA isolation and reverse transcription were carried out according to the previously published method [5]. Fold change was calculated as described earlier [18]. The gene-specific primers were purchased from Qiagen (USA).

### 2.4. Mitochondrial Electron Transport Chain Complexes

Activities of mitochondrial electron transport chain complex I, complex II, and complex IV were performed (Mitosciences, USA), and activities were expressed as fold change as described earlier [5]. Activities were determined from total tissue lysates as recommended by the manufacturer.

### 2.5. Liver Oxidative Stress Markers

Liver 3-NT levels were determined with ELISA kit from Hycult Biotechnology, Cell Sciences, Canton, USA, as described earlier [5].

HNE adducts were determined using the OxiSelect™ HNE Adduct ELISA Kit according to manufacturer's instruction (Cell Biolabs, Genetimes Technology Inc., Shanghai, China) as described earlier [19].

### 2.6. Nuclear and Cytoplasmic Fractionation

Nuclear and cytoplasmic fractions were separated from the same set of samples by commercial kit (NE-PER Nuclear and Cytoplasmic Extraction Reagents, Thermo Fisher Scientific, USA).

### 2.7. Mitochondria Isolation

Mitochondria were isolated using commercial tissue mitochondria isolation kit (Pierce, USA) as described earlier [5].

### 2.8. Immunoblot Analyses

The protein contents were determined according to the Bradford method. Aliquots of 40 micrograms of protein were analyzed by SDS-PAGE. Western blots were carried out using antibodies for TNF*α*, GAPDH, NRF2, and lamin A (Abcam Company Ltd., China), Secondary antibodies were purchased from Fazendo Media (Beijing, China).

### 2.9. Liver PARP Activity

Liver PARP activities were determined using the commercial assay kit (Trevigen Inc.) as described earlier [5]. The quantitative values were expressed as fold change compared to those of the sham control.

### 2.10. Liver Apoptotic Markers

Caspase 3 activities were measured by using colorimetric caspase 3 activity assay kit (Beyotime Biotech, China) according to the manufacturer's instruction and expressed as fold change. DNA fragment quantifications were performed with the DNA fragmentation kit (Takara, China) according to the manufacturer's protocol.

### 2.11. Stellate Cell Isolation and Culture

Primary stellate cells from mice were isolated based on our earlier published method [5]. Primary stellate cells were grown for 8 days. Baicalin was added at 20 *μ*M every day after replacing with fresh media. RNA was extracted either from fresh culture (day 0) or after 8 days (day 8), and real-time PCR was performed after reverse transcription.

### 2.12. Statistical Analysis

All graphical data were shown as the means ± SEMs. Multiple comparisons were performed using one-way ANOVA using Graph Pad Prism software (USA). A *p* value less than 0.05 was considered statistically significant.

## 3. Results and Discussions

### 3.1. Baicalin Attenuates BDL-Induced Liver Fibrosis in Mice

**Figure 2:**
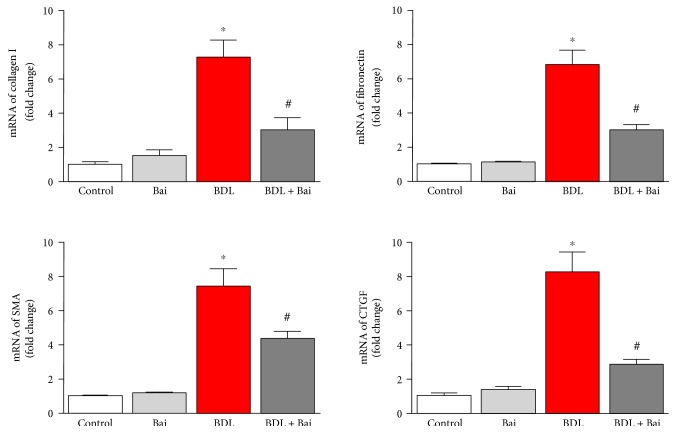
Baicalin markedly attenuated BDL-induced fibrotic gene expression. BDL induced liver profibrotic genes at mRNA level. Real-time PCR analyses were performed for collagen I, fibronectin, alpha smooth muscle actin (SMA), and CTGF genes. BDL-induced increases in all four gene expressions and the inductions were significantly reduced by baicalin treatment. Results are mean ± S.E.M. *n* = 6 for each group. ^∗^*p* < 0.05 versus control and ^#^*p* < 0.05 versus BDL.

BDL for 15 days induced significant fibrosis based on picosirius red staining, which stains collagen proteins in parenchymal cells (Figure 1(b)). Treatment with baicalin at 200 mg/kg dose on alternate day reduced fibrosis. Image quant analysis of the images demonstrated that 17.9-fold increased in fibrotic area was observed due to BDL and baicalin attenuated up to 40.3% (Figure 1(c)). In addition to fibrosis, significant increase of hepatocyte necrosis was observed and baicalin treatment attenuated those necrosis incidents (Figure 1(d)). To understand the molecular basis of fibrosis, we determined mRNA level of genes associated with fibrosis by real-time PCR. Collagen I, fibronectin, SMA, and CTGF were induced to 7.3-, 6.8-, 7.5-, and 8.3-fold, respectively, in BDL model (Figure 2()). Alternate day of baicalin treatment attenuated BDL-induced mRNA of collagen I, fibronectin, SMA, and CTGF to 58%, 55%, 42%, and 65% of BDL level, respectively.

We demonstrated that baicalin protected against liver fibrosis in a well-established mice of BDL. Baicalin is the glucuronide of baicalein, which is obtained through the binding of glucuronic acid to baicalein. Baicalin has both antioxidant and anti-inflammatory properties [20, 21]. The metabolism of baicalin was studied in rodent model and no toxicity was observed [22, 23]. Liver fibrosis induced by BDL has the deposition of connective tissue elements and formation of ductular followed by necrosis [24]. Role of hepatocytes are crucial in development of liver fibrosis. Cell death of hepatocytes leads to a series of proinflammatory response [25]. Hepatocyte has complex role in liver injuries and multiple factors regulate the process including retinoic acid [26]. It is also known that biliary epithelial cells and hepatocytes can undergo epithelial–mesenchymal transition during liver fibrosis [27]. Expression of profibrotic markers such as collagen I, fibronectin, SMA and CTGF in response to BDL has been reported earlier [5, 18]. We demonstrated that all profibrotic markers were attenuated by alternate day treatment of baicalin. Consistent with our study, the protective effect of baicalin in other mouse models of liver fibrosis has been reported [28].

### 3.2. Baicalin Ameliorates BDL-Induced Liver Inflammation in Mice

**Figure 3:**
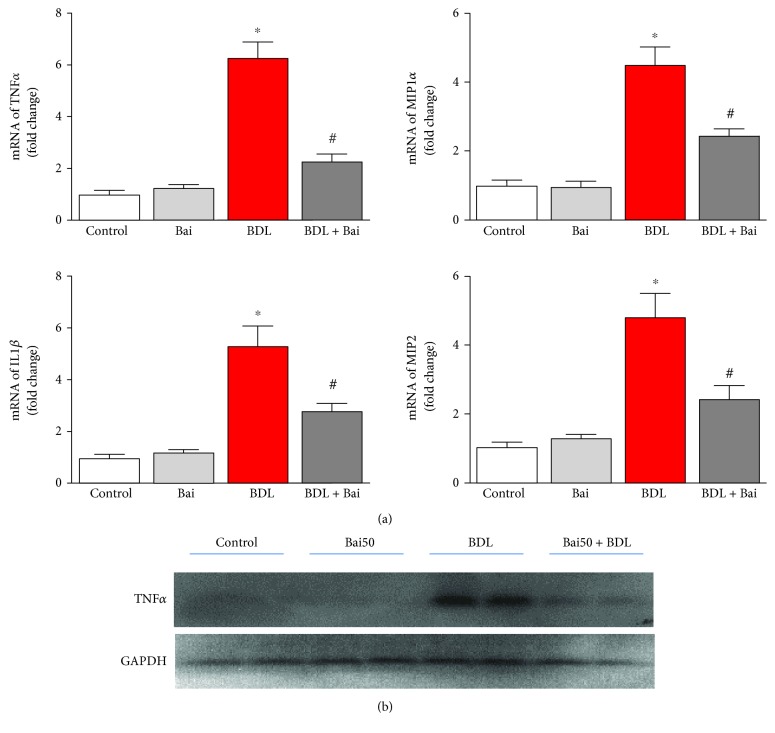
Effect of baicalin on BDL-induced proinflammatory cytokines in mice liver. (a) BDL-induced proinflammatory cytokines as measured by real-time PCR analyses for TNF*α*, MIP1*α*, IL1*β*, and MIP2 genes. BDL-induced proinflammatory cytokine mRNA was attenuated by alternate day baicalin treatment. Results are mean ± S.E.M. *n* = 6 for each group. ^∗^*p* < 0.05 versus control and ^#^*p* < 0.05 versus BDL. (b) Protein level of TNF*α* was verified by immunoblot analyses, and GAPDH was used as the loading control.

BDL induced fibrosis, which is initiated by cell death-associated inflammatory stimuli. We determined proinflammatory cytokines TNF*α*, MIP1*α*, IL1*β*, and MIP2 by real-time PCR. The result demonstrated induced TNF*α*, MIP1*α*, IL1*β*, and MIP2 mRNA levels to 6.29-fold, 4.51-fold, 5.32-fold, and 4.83-fold, respectively (Figure 3(a)). Administration of baicalin on alternate day for 15 days reduced BDL-induced proinflammatory cytokines TNF*α*, MIP1*α*, IL1*β*, and MIP2 to 63%, 47%, 47%, and 49% respectively. To verify the mRNA signal to protein level, we examined one marker TNF*α* by immunoblot analyses and BDL increased TNF*α* protein level and was attenuated by baicalin, which is consistent with the mRNA expression pattern.

Our study demonstrated that baicalin prevents fibrosis by attenuating BDL-induced proinflammatory cytokines and inflammation. Modulation of inflammatory signal by baicalin in the liver for hepato-protection is well known in other liver injuries induced by concanavalin A, D-galactosamine, and alcoholic fatty liver [13, 14, 29]. The resident macrophage, known as the Kupffer cell, plays critical role in this process [30].

### 3.3. Baicalin Ameliorates BDL-Induced Oxidative Stress Markers in Mice

**Figure 4:**
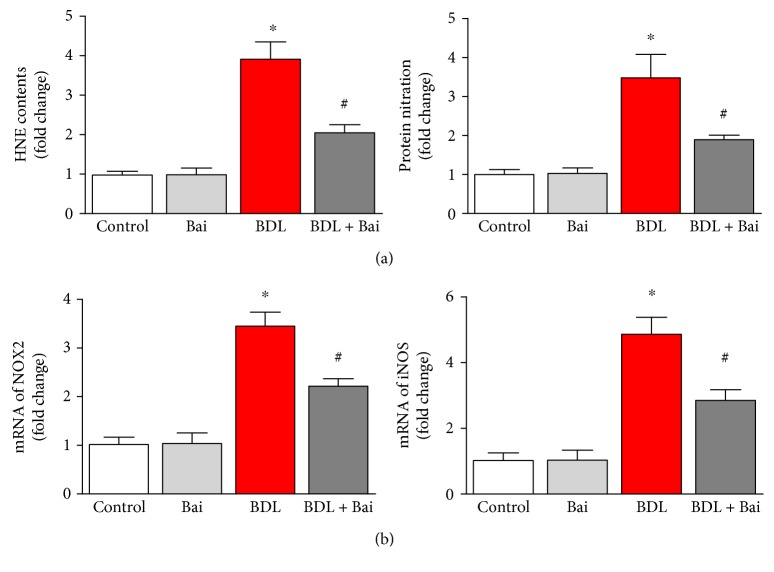
Baicalin significantly reduced BDL-induced oxidative/nitrative stress in mouse liver. BDL caused significant increase in oxidative and nitrative stress as measured by HNE protein adducts and protein nitration using commercial ELISA kits. BDL induced nitrative stress and oxidative stresses (a) and was attenuated by baicalin treatment. Results are mean ± S.E.M. *n* = 6 for each group. ^∗^*p* < 0.05 versus control and ^#^*p* < 0.05 versus BDL. Real-time PCR analyses were performed for NOX2 and iNOS genes (b). BDL induced both gene expression, and the inductions were significantly reduced by baicalin treatment. Results are mean ± S.E.M. *n* = 6 for each group. ^∗^*p* < 0.05 versus control and ^#^*p* < 0.05 versus BDL.

Inflammatory response in BDL induces oxidative stress and reduces cellular antioxidant capability. Here, we determined oxidative/nitrative markers and their causes. HNE protein adducts increased significantly in the liver due to BDL (3.9-fold) and baicalin treatment reduced significantly to 47% (Figure 4(a)). Hepatic protein nitration is also increased to 3.5-fold and baicalin attenuated 45.7% from BDL (Figure 4(a)). The sources of oxidative stress were investigated by real-time PCR of NOX2 and iNOS. Both of them were induced in BDL and attenuated by baicalin. Treatment with baicalin in control group does not have any effect of oxidative stress and ROS-generating sources.

BDL induced significant oxidative stress through bile salt and other free radicals which causes damage to other organs such the kidney in addition to liver [31]. In liver injury, hepatocytes are primary parenchymal cells targeted by oxidative stress [32]. Consistent with our study in BDL, the antioxidant properties of baicalin are reported in many other liver injury models [28, 33].

### 3.4. Baicalin Improves BDL-Impaired Mitochondrial Membrane Complex Activities in Mice

**Figure 5:**
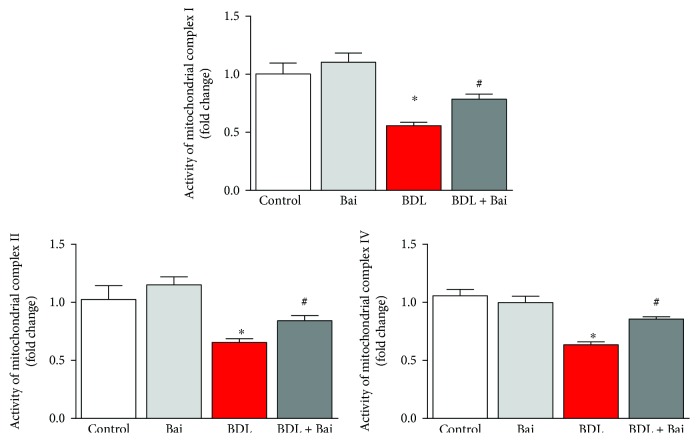
Baicalin improved BDL-induced decrease of mitochondrial membrane complex activities in mouse liver. BDL caused decrease in mitochondrial membrane complexes as measured from enzyme activities of electron transport chain complex I, complex II, and complex IV. BDL mediated decrease of mitochondrial complex activities were improved level by alternate day of baicalin treatment. Results are mean ± S.E.M. *n* = 6 for each group. ^∗^*p* < 0.05 versus control and ^#^*p* < 0.05 versus BDL.

Mitochondrial electron transport chain is one of the major sources for free radical generation and oxidative injury. We examined electron transport chain complex activities from isolated mitochondria of the liver. BDL surgeries in mice reduced complex I, complex II, and complex IV activities to 45%, 35%, and 37%, respectively (Figure 5). Administration of baicalin every day during two weeks BDL improved complex I activity to 40%, complex II to 31%, and complex IV to 33%. Thus, baicalin administration significantly ameliorates BDL-induced mitochondrial dysfunction and associated oxidative injury in the liver.

Mitochondria play a bigger role in BDL-induced liver injury [34, 35]. The dysfunctional electron transport chain in mitochondria is one of the major sources of ROS [36]. We observed dysfunctional electron transport chain in BDL mouse liver, and baicalin improved those functions of electron chain complexes. Baicalin ameliorates mitochondrial damage in streptozotocin-induced diabetic rats, and its beneficial effects on mitochondria are reported [37–39]. Baicalin has a beneficial effect on mitochondrial function [38].

### 3.5. Baicalin Enhances BDL-Induced Nuclear Translocation of Nrf2 and Increases HO-1 and GCLM Expression

**Figure 6:**
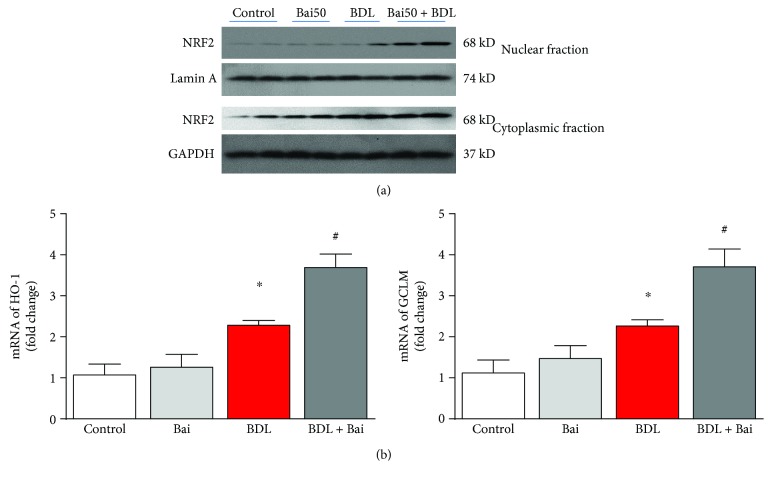
Baicalin enhanced nuclear translocation of NRF2 in BDL mice and increased transcription of its target genes HO-1 and GCLM. (a) Western blot images showing BDL-induced nuclear localization of NRF2 were further enhanced significantly by baicalin treatment. Western blot analyses of cytoplasmic fraction showing increased expression of NRF2 in samples with BDL pretreated with baicalin. (b) Real-time PCR analyses were performed for HO-1 and GCLM genes. BDL induced both gene expressions, and the inductions were significantly enhanced by baicalin treatment. Results are mean ± S.E.M. *n* = 6 for each group. ^∗^*p* < 0.05 versus control and ^#^*p* < 0.05 versus BDL.

NRF2 is key transcriptional regulator for antioxidant defense in many liver diseases [40]. Baicalin significantly enhanced nuclear localization of NRF2 (Figure 6(a), top panel) in BDL mice. An increase in BDL-induced NRF2 nuclear localization may be due to induction of endogenous antioxidant defense in response to oxidative stress but not sufficient to protect against injury. In addition, we also observed little increase of NRF2 in cytoplasmic fractions of BDL mice pretreated with baicalin (Figure 6(a), bottom panel). These results indicated baicalin mediated upregulation of NRF2 in fibrotic liver. Nuclear translocation of NRF2 is also associated with transcriptional activation as evident by the fact that the target genes (HO-1 and GCLM) also induced in the same pattern as nuclear translocation (Figure 6(b)).

The hepatoprotective effect of many naturally occurring compounds is mediated by NRF2 [41]. NRF2 is also is a key regulator of oxidative stress in numerous cell types [42, 43]. In BDL, Nrf2-deficient mice show reduced elimination of bile acids leading to liver injury [44]. Consistent with the key role of NRF2 in BDL, we also demonstrated that baicalin modulates NFR2 and its increased nuclear translocation led to protective effect. The induction of antioxidant defense such as HO-1 by baicalin-induced NRF2 translocation may be responsible for hepatoprotection. NRF2 translocation by baicalin is not mediated by any pro-oxidant properties, but the modulation is at the molecular level. This hypothesis is further supported by the fact that baicalin did not induce NRF2 in the control group.

### 3.6. Baicalin Ameliorates BDL-Induced Cell Death Pathway in Mouse Liver

**Figure 7:**
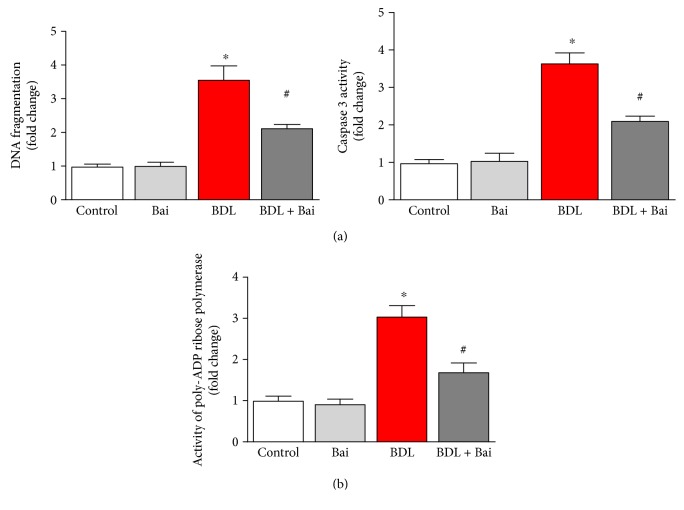
Baicalin markedly attenuated BDL-induced apoptotic and necrotic cell death markers DNA fragmentation, caspase 3 activity, and PARP activity in mouse liver. BDL induced significant increases in liver DNA fragmentation and caspase 3 activity, both markers of apoptotic cell death (a). BDL induced significant increase in PARP activity, a marker of necrotic and apoptotic cell death (b). BDL-induced increases in all three cell death markers were attenuated by baicalin treatment. Results are mean ± S.E.M. *n* = 6/group. ^∗^*p* < 0.05 versus control and ^#^*p* < 0.05 versus BDL.

BDL induces significant apoptotic and necrotic cell death in liver [5]. We examined three cell death markers DNA fragmentation, PARP activity, and caspase 3 activity. BDL induced 3.6-fold and 3.7-fold increase in apoptotic cell death markers DNA fragmentation and caspase 3 activity. Baicalin administration reduced BDL-induced DNA fragmentation and caspase 3 activity up to 40% and 42%, respectively (Figure 7(a)). We also investigated another cell death marker PARP activity which represents necrotic pathway and apoptotic pathways in liver injury [6]. BDL induced 3-fold increase in PARP activity, and baicalin administration significantly reduced BDL-induced PARP activity (44.5%) (Figure 7(b)).

Oxidative stress is major source for cell death in BDL [45, 46]. Intervention of PARP is shown to ameliorate liver fibrosis in BDL [4]. Baicalin prevented BDL-induced apoptotic and necrotic cell death. The effect may be due to reducing oxidative stress, promoting antioxidant defense, and attenuating inflammation.

### 3.7. Mechanistic Role of Stellate Cells in Baicalin-Mediated Antifibrotic Action

**Figure 8:**
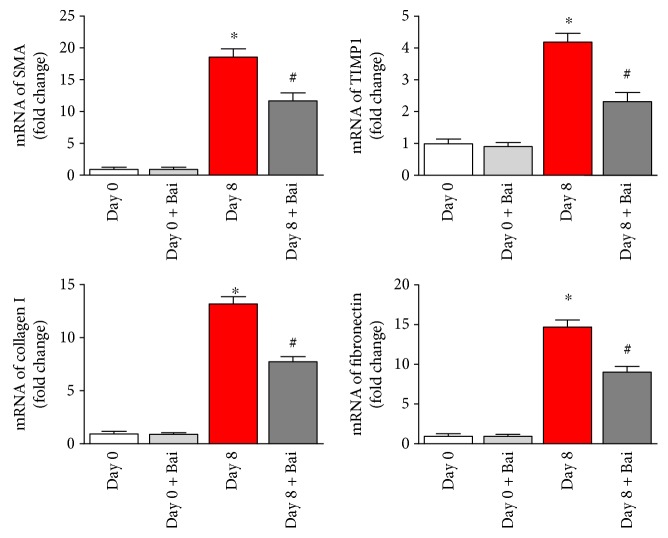
Baicalin attenuated profibrotic gene expression in isolated mouse stellate cells. Stellates were cultured for 8 days to stimulate activation in culture and compared with fresh isolated stellate cells (day 0). Real-time PCR analyses of profibrotic mRNA of smooth muscle actin alpha (SMA), TIMP1, collagen 1, and fibronectin were induced during fibrosis stimulation. Baicalin reversed mRNA expressions in all four genes. Results are mean ± S.E.M. *n* = 4 for each group. ^∗^*p* < 0.05 versus day 0 and ^#^*p* < 0.05 versus day 8.

Hepatic stellate cells contribute to liver fibrosis through various steps including extracellular matrix homeostasis [47]. As we observed significant drop in liver fibrosis by baicalin, an in vitro study with stellate cells was essential. We used a natural phenomenon where spontaneous activation occurs at day 8 when stellate cells grow under in vitro condition [5]. To elucidate the inhibitory role of baicalin in the activation of stellate cell process and its effect on profibrotic marker production, we examined using real-time PCR analyses. The data demonstrated that baicalin treatment at 20 *μ*M significantly reduced profibrotic genes such as SMA, collagen 1, and fibronectin (Figure 8).

After liver injury, stellate cell activation plays central role in initiation and propagation of liver fibrosis [48]. Kupffer cell activation facilitates stellate cell activation by secretion of TGF*β* and ROS in the extracellular environment [30]. We observed modulation of stellate cell activation by baicalin in vitro. This is significant because of the fact that baicalin can modulate liver fibrosis not only by reducing oxidative stress/inflammation/cell death but also by regulating stellate cell activation.

## 4. Conclusion

**Figure 9:**
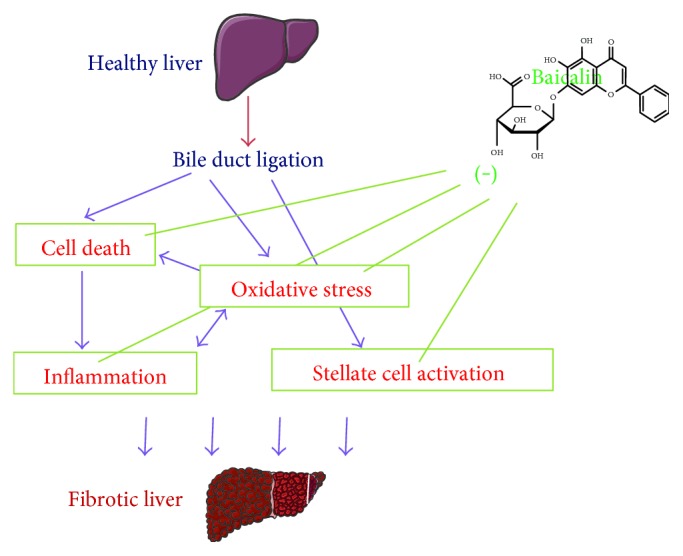
Schematic diagram of baicalin-mediated protection in experimental cholestatic liver injury. Bile duct ligation of healthy liver leads to hepatocyte cell death and oxidative stress. Both of these factors lead to proinflammatory responses, and significant inflammation occurs. Inflammation also leads to increased oxidative stress and thus interplay each other. Baicalin protects against cell death, oxidative stress, and inflammation. In addition to that, stellate cell activation occurs which leads to liver fibrosis. Under in vitro condition, baicalin attenuates stellate cell activation. All the above pathophysiological conditions lead to fibrotic liver.

The complexities of liver fibrosis and lack of approved treatment to patients lead this subject to forefront to researchers and clinicians. In our study, baicalin partially protects against experimental liver cholestasis via multiple mechanisms (Figure 9). The complexities of cholestatic liver fibrosis are due to the involvement of different cells in the liver such as hepatocytes, stellate cells, and immune cells, and their interactions are real crucial. Reversal of such process is challenging. Thus, the limitation of baicalin-mediated protection is due to the additional challenging factors including bioavailability, selective transportation to hepatocytes or stellate cells, or targeting other cell types. Despite the above limitations, baicalin protects against experimental cholestatic liver fibrosis by reducing inflammation, oxidative stress, and cell death and enhancing antioxidant defense in addition to modulating stellate cell activation.
